# Association of baseline self-reported fatigue with overall survival after stereotactic body radiation therapy for localized prostate cancer

**DOI:** 10.3389/fonc.2022.1015264

**Published:** 2022-12-23

**Authors:** Rishabh K. Simhal, Tamir N. Sholklapper, Anish K. Simhal, Alan L. Zwart, Malika T. Danner, Deepak Kumar, Nima Aghdam, Simeng Suy, Ryan A. Hankins, Keith J. Kowalczyk, Sean P. Collins

**Affiliations:** ^1^ School of Medicine, Georgetown University, Washington, DC, United States; ^2^ Department of Medical Physics, Memorial Sloan Kettering Cancer Center, New York, NY, United States; ^3^ Department of Radiation Oncology, MedStar Georgetown University Hospital, Washington, DC, United States; ^4^ Julius L. Chambers Research Institute, North Carolina Central University, Durham, NC, United States; ^5^ Department of Radiation Oncology, Beth Israel Deaconess Medical Center, Boston, MA, United States; ^6^ Department of Urology, MedStar Georgetown University Hospital, Washington, DC, United States

**Keywords:** prostate cancer, SBRT (stereotactic body radiation therapy), overall survival, baseline fatigue, quality of life, prostate radiation therapy, localized prostate cancer (PCa), urology

## Abstract

**Introduction:**

Stereotactic Body Radiation Therapy (SBRT) has emerged as a definitive therapy for localized prostate cancer (PCa). However, more data is needed to predict patient prognosis to help guide which patients will benefit most from treatment. The FACIT-Fatigue (FACIT-F) is a well validated, widely used survey for assessing fatigue. However, the role of fatigue in predicting PCa survival has yet to be studied. Herein, we investigate the role of FACIT-F as a baseline predictor for overall survival (OS) in patients undergoing SBRT for localized PCa.

**Methods:**

A retrospective review was conducted of 1358 patients who received SBRT monotherapy between January 2008 to April 2021 at an academic, tertiary referral center. FACIT-F scores (range 0 to 52) were summed for patients who answered all 13-items on the survey. FACIT-F total scores of ≥35 represented severe fatigue. Patients receiving androgen deprivation therapy were excluded. Differences in fatigue groups were evaluated using chi-squared tests. OS rates were determined using the Kaplan-Meier method and predictors of OS were evaluated using Cox proportional hazard method.

**Results:**

Baseline full FACIT-F scores and survival data was available for 891 patients. 5-year OS was 87.6% and 95.2%, respectively, for the severely fatigued and non-fatigued groups. Chi-squared analysis of fatigue groups showed no significant difference in the following categories: D’Amico risk group, age, ethnicity, grade group, T-stage, or PSA density. Severe fatigue was associated with a significant decrease in OS (hazard ratio 2.76; 95%CI 1.55 - 4.89). The Cox proportional hazard model revealed that age and FACIT-F were both statistically significant (p <0.05).

**Conclusion:**

Baseline FACIT-F scores are significantly associated with OS. Higher FACIT-F scores, representing less fatigued patients, are associated with an overall survival benefit. These results indicate that the FACIT-F survey could serve as an additional metric for clinicians in determining prognostic factors for patients undergoing SBRT.

## Introduction

Prostate cancer is the most common cancer in men worldwide. Elderly men are commonly treated with various forms of radiation therapy that may cause bothersome side effects (1). Men with life expectancies of less than 10 years may not benefit from such treatment (2). Hence, estimation of a given patient’s future survival is important in the decision-making process between watchful waiting and radiation therapy. Previously, predictors of mortality have been reported such as older age at diagnosis, performance status, comorbidities, socioeconomic status, and race, amongst others (3–5). Life expectancy is currently determined using Social Security Administrative tables or available life expectancy tools generated by combining several of the known prognostic variables (6). However, current models are not perfect and may be overly complicated for use in a busy clinic. A clinician’s judgment of a patient’s overall health may be useful in adjusting an individual patient’s predicted life expectancy (7).

Stereotactic body radiation therapy (SBRT) has recently emerged as more accurate, convenient and cost-effective definitive radiation modality for prostate cancer (8). Given these benefits, as well as the emerging literature showing comparable oncological outcomes to patients treated with conventional fractionated radiotherapy (EBRT), SBRT for prostate cancer has become more widely adopted (9, 10). Treatment with SBRT however is not without morbidity itself, and patients diagnosed with prostate cancer must make the decision along with clinicians to determine if SBRT is justified for an individual patient.

Baseline quality of life (QoL) metrics can predict survival in prostate cancer patients and could add to the factors that guide whether patients should undergo treatment (11). Self-reported fatigue is frequently measured in cancer patients and is a useful QoL metric for clinicians to monitor. The Functional Assessment of Chronic Illness Therapy Fatigue (FACIT-F) is a 13 point patient reported outcome questionnaire designed to assess a patient’s fatigue and its impact on daily functioning. FACIT-F has been widely used in the literature for decades, and has been well validated in a variety of neoplastic and non-neoplastic diseases (12–14). A recent study on high-grade glioma patients found that FACIT-F scores were strong independent predictors of survival, with patients who were severely fatigued (FACIT-F < 35) had statistically worse survival outcomes compared to the less fatigued group (FACIT-F > 35) (15). Of note, in this cohort of high-grade glioma patients, the disease and treatment of it profoundly affect fatigue, whereas in prostate cancer, fatigue may be a more subtle predictor of outcomes. Other survey tools have also tried to assess fatigue. The Expanded Prostate Cancer Index Composite for Clinical Practice (EPIC-CP) is a 12 question multidomain survey looking at multiple aspects of quality of life in prostate cancer patients such including fatigue, and has been validated and widely used in prostate cancer patients (16, 17). However, to date no studies have looked at EPIC-CP in SBRT patients in regards to fatigue. In this retrospective cohort study, we aim to investigate if fatigue, as measured by FACIT-F scores, is a predictor of survival in patients being treated by SBRT for prostate cancer.

## Materials and methods

### Patient selection

Institutional IRB approval was obtained for retrospective review of from our institutional database (IRB#: 2009-510). Patients eligible for this study were those who had histologically confirmed prostate cancer. Patients who received androgen deprivation therapy (ADT) were excluded from this study as this represented major confounder in analyzing both fatigue prior to and survival after SBRT. Patients treated from 2008 to 2021 were included.

### SBRT treatment planning and delivery

SBRT treatment planning and delivery were conducted as previously described (18). Briefly, gold fiducials were placed into the prostate. Fused CT and MR images were used for treatment planning. The clinical target volume (CTV) included the prostate and the proximal seminal vesicles (to the point where the seminal vesicles separate). The planning target volume (PTV) equaled the CTV expanded 3 mm posteriorly and 5 mm in all other dimensions. The prescription dose was 35-36.25 Gy to the PTV delivered in five fractions of 7-7.25 Gy over one to two weeks.

### FACIT-Fatigue

Patient-reported outcomes were obtained at initial consultation, the first day (start) of treatment. Fatigue was assessed via the Functional Assessment of Chronic Illness Therapy -Fatigue (FACIT-F).

The FACIT-F is a 13-item subscale (https://www.facit.org/measures/FACIT-F) of the 47 question Functional Assessment of Cancer Therapy – Anemia (FACT-An). This scale was developed nearly 3 decades ago and has been validated in numerous populations with chronic ailments. The FACIT-F was scored per the guidelines listed on www.facit.org and ranged from 0 – 52 with higher scores representing better quality of life (QOL). A cutoff of ≤35 was used as a cut off score for clinically significant fatigue as this score has previously been found to be associated health-related QOL outcomes (15, 19, 20).

### Statistical analysis

Baseline patient and disease characteristics were analyzed with sample medians and interquartile ranges were used to describe continuous variables. The baseline patient characteristics that were included: age, race, body mass index (BMI), biopsy grade group, T-stage, D’Amico risk group, prostate specific antigen (PSA), and testosterone level. Differences among those with or without clinically significant fatigue (≤35 or >35, respectively) were analyzed *via* χ2, Fisher’s exact, or Student’s t-test, as appropriate (21).

Survival was analyzed via Kaplan-Meier method with predictors of OS, including FACIT-F, were evaluated using multivariate Cox proportional hazard method. For all analysis, missing data were excluded by default. All tests were two-tailed, and a p-value <0.05 was considered significant. JMP^®^ PRO version 15.0.0 was used to perform the statistical analyses (SAS Institute Inc., Cary, NC, 1989-2019).

## Results

Of the 1,358 patients treated with SBRT, survival data were available for 1,007 patients who were treated with SBRT-monotherapy with a median follow up time of 43 months. Of these patients, 891 patients had completed the FACIT-F questionnaire at baseline. **Table 1** includes a summary of baseline patient and treatment characteristics with a comparison between those with or without clinically significant fatigue. Median age was 69 (interquartile range [IQR] 64 - 74) years old. The majority of patients were either White or Hispanic (59.7% and 35.2%, respectively). The majority of patients had biopsy-confirmed, grade group 2 or 1 disease (44.9% and 32.1%, respectively) and were classified as intermediate or low D’Amico risk-group (73.8% and 21.6%, respectively). Clinically significant fatigue (FACIT-F ≤35) was present in 12.7% of patients. There were statistically significant differences in fatigue groups by race, BMI, and baseline testosterone levels (*p*=0.048, *p*=<0.001, and *p*=0.040 respectively). There was no clinically significant difference by age at treatment, grade group, T-stage, D’Amico risk group, or PSA level (*p*>0.05).

**Table 1:** Baseline patient, and treatment characteristics.

	FACIT Fatigue				
	≤ 35		> 35		
	n	(%)	n	(%)	*p*-value
Patients	113		778		
Age					0.9032
Median (IQR), y	68	(65, 74)	69	(64, 74)	
≤65	35	(31.0%)	235	(30.2%)	
66-75	62	(54.9%)	400	(51.4%)	
>75	16	(14.2%)	143	(18.4%)	
Race/Ethnicity					**0.0478**
White or caucasian	56	(49.6%)	476	(61.2%)	
Black or AA	50	(44.2%)	264	(33.9%)	
Hispanic	1	(0.9%)	13	(1.7%)	
Asian	6	(5.3%)	19	(2.4%)	
Other	0	(0.0%)	6	(0.8%)	
BMI					**0.0004**
Median (IQR), kg/m^2	29.7	(26.5, 33.1)	27.4	(25.0, 30.9)	
<25	11	(10.4%)	186	(24.8%)	
25-29.9	45	(42.5%)	337	(45.0%)	
≥30	50	(47.2%)	226	(30.2%)	
EPIC Fatigue					**<.0001**
No problem to small problem	56	(49.6%)	765	(98.3%)	
Moderate to big problem	57	(50.4%)	13	(1.7%)	
Grade group (gleason)					0.4386
1 (3 + 3)	32	(28.6%)	253	(32.6%)	
2 (3 + 4)	52	(46.4%)	347	(44.7%)	
3 (4 + 3)	24	(21.4%)	158	(20.3%)	
4 (4 + 4)	4	(3.6%)	13	(1.7%)	
5 (4 + 5 or 5 + 5)	0	(0.0%)	6	(0.8%)	
T-stage					0.6035
T1-T2a	99	(88.4%)	661	(85.8%)	
T2b-c	13	(11.6%)	107	(13.9%)	
T3	0	(0.0%)	2	(0.3%)	
Risk Group, D'Amico					0.6290
Low	22	(19.5%)	170	(21.9%)	
Intermediate	84	(74.3%)	572	(73.7%)	
High	7	(6.2%)	34	(4.4%)	
PSA, ng/dL					0.3897
Median (IQR), y	7.8	(5.3, 12)	7.1	(5.4, 10)	
<10	71	(17.4%)	575	(23.1%)	
10 - 20	39	(72.9%)	177	(72.1%)	
>20	3	(9.7%)	26	(5.6%)	
PSA Density, ng/mL					0.1933
≤0.15	30	(27.5%)	262	(34.0%)	
>0.15	79	(72.5%)	508	(66.0%)	
Testosterone, ng/dL					
Median (IQR), y	341.0	(209, 459)	363.0	(277, 483)	**0.0404**

AA, African American. Bold values indicate results that are statistically significant, with p-values less that 0.05.

**Figure 1:**
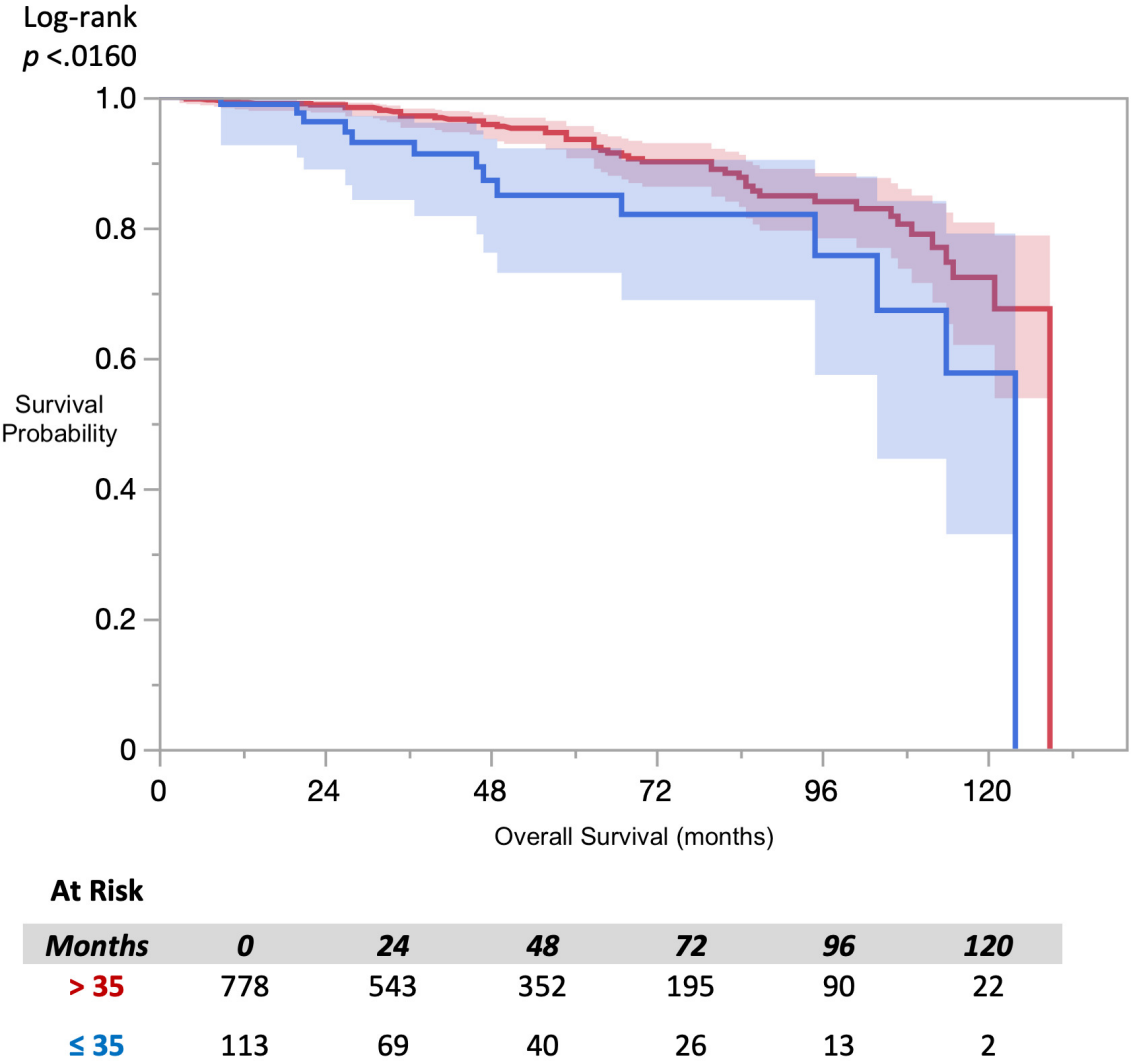
KM Overall Survival FACIT- Fatigue.

Analysis of overall survival using the Kaplan-Meier method (**Figure 1**), revealed a statistically significant difference in survival by FACIT-F groupings (log-rank *p*=0.016). Multivariate Cox-proportional hazard analysis of FACIT-F (**Table 2**), adjusted for age, race, and D’Amico risk group,

**Table 2:** Multiple regression hazard analysis FACIT-F.

	HR	(95% CI)	p-value
Age			
≤65	ref		
66-75	1.59	(0.81, 3.14)	0.1775
>75	3.04	(1.38, 6.68)	**0.0056**
Race/Ethnicity			
White or caucasian	ref		
Black or AA	1.31	(0.76, 2.25)	0.3279
Hispanic	1.51	(0.20, 11.26)	0.6852
Asian	0.54	(0.07, 4.03)	0.5504
Other	N/A		
Risk Group, D'Amico			
Low	ref		
Intermediate	1.79	(0.87, 3.68)	0.1156
High	3.65	(1.37, 9.72)	**0.0096**
Body Mass Index (BMI)			
< 25	ref		
25 - 29.9	1.01	(0.52, 1.99)	0.9704
> 30	0.80	(0.36, 1.75)	0.5691
Testosterone			
Range Risk Ratio	2.21	(0.45, 10.91)	> 0.05
Baseline FACIT-Fatigue			
≤ 35	2.16	(1.18, 3.93)	**0.0121**
> 35	ref		

Bold values indicate results that are statistically significant, with p-values less that 0.05.

(95% CI 1.18-3.93, *p*=0.012) in patients with clinically significant fatigue compared to those without fatigue at baseline. There were statically significant increased in likelihood of mortality events in patients over 75 years old (HR 2.81, 95% CI 1.34 - 5.92, compared those ≤65) and patients classified at high-risk (HR 3.92, 95% CI 1.57 - 9.79, compared to low-risk); however, there was no statistically significant difference in this likelihood for patients aged 66-75 years and patients with intermediate-risk, respectively.

## Discussion

In this large retrospective cohort study, baseline fatigue was found to be correlated with overall survival in patients undergoing SBRT for localized prostate cancer. Patients who were more fatigued prior to treatment had worse survival outcomes compared to their less fatigued peers. This correlation was present when fatigue was measured using the FACIT-F questionnaire, an in depth fatigue questionnaire that has been widely used to measure fatigue in cancer patients. This correlation may be a helpful addition to the standard predictors of overall survival when discussing treatment options with patients and having conversations guided by shared decision-making principles.

Given our findings, we hypothesize that baseline fatigue may be a reflection of overall patient health status, possibly related to medical comorbidities not captured in this analysis. Numerous studies have shown the association of fatigue with disease severity of other medical conditions such as depression and autoimmune disease (22, 23). Therefore, self-reported fatigue may not only serve as a proxy for medical comorbidities, but uniquely effective as it may also capture the severity of those comorbidities. Common medical comorbidities and baseline patient demographics have recently been employed in other prostate cancer risk calculators, however this study is unique in how we report the predictive value of a self-reported characteristic (24). Risk calculators that rely on common medical comorbidities are inherently limited by only being able to predict based off the few common medical comorbidities captured in the calculator. Given fatigue may be reflective of disease severity across numerous domains of medical comorbidities, it may be helpful in predicting survival and warrants further investigation as a potential factor to be included into other mortality calculators.

One concern about utilization of a one-time questionnaire in guiding treatment decisions is that it might not accurately reflect the patient’s normal condition. Psychometric assessment has demonstrated that the FACIT fatigue scale is stable overtime with excellent test-retest reliability (12, 14). Moreover, even within our dataset, FACIT scores remained relatively stable for patients across the baseline pretreatment visits as well as into the first few treatment visits. It is also reassuring that there was also a statistically significant correlation between FACIT-F scores and the fatigue subsection of the EPIC-CP questionnaire, which is a single question asking patients to rate how much of a problem fatigue has been for the last 4 weeks on a scale of 0 to 4. The EPIC-CP questionnaire has been widely adopted in both urology and radiation oncology practices as an effective tool for measuring symptoms from prostate cancer and subsequent prostate cancer treatments, and has even shown utility in helping guide treatment modalities with shared decision making principles (17, 25, 26). This makes measuring fatigue a more practical clinical tool, as although the FACIT-F is not regularly used in urology clinics, the EPIC-CP questionnaire is exceedingly common in urooncology practices. Given this correlation, future studies should assess to see if there is a direct correlation between the EPIC fatigue score and overall survival.

A unique strength of this study was the diversity of the dataset - 35% of the patients identified as Black or African American. Data that are applicable to the Black and non-Black population is helpful, as numerous studies have shown the increased incidence and severity of prostate cancer in the Black population (27, 28). Given disparities in health outcomes by race, clinicians should be mindful of the representation within the data sets of studies they refer to when counseling patients.

This study is not without its limitations however. There was limited survival data, and our database did not include patients’ comorbidities prior to treatment. Therefore, other medical comorbidities may be correlated with fatigue and act as a potential confounder. However, despite the potential for this confounder, there is still independent clinical utility to the ease at which fatigue can be assessed on a single point questionnaire. Calculating a Comorbidity Index such as the ACE27 for example (29), is often cumbersome to specialist clinicians, as it often requires in depth review of a patient’s chart and communication with a patient’s primary care provider. Therefore, a unique strength of this correlation with fatigue and survival is that despite a potential confounding effect with medical comorbidity, calculating a fatigue score can be significantly more efficient and accessible in an outpatient clinical setting. However, future studies should include medical comorbidity data when analyzing the predictive value of baseline fatigue with overall survival.

Continued analysis of the dataset as more survival data becomes available may also be useful to continue to investigate this relationship. Other future directions to investigate fatigue and overall survival is to expand the study to include patients beyond SBRT to include other types of radiotherapy such as EBRT. This study could also be further expanded to prostate cancer patients undergoing other cornerstone treatment modalities such as radical prostatectomy or active surveillance.

## Data availability statement

The data analyzed in this study is subject to the following licenses/restrictions: Dataset maintained by MedStar Georgetown University Hospital. Requests to access these datasets should be directed to RS, rishi.simhal@gmail.com.

## Ethics statement

The studies involving human participants were reviewed and approved by MedStar Georgetown University Hospital Institutional Review Board IRB#: 2009-510. Written informed consent for participation was not required for this study in accordance with the national legislation and the institutional requirements.

## Author contributions

RS and TS contributed equally to this project. RS, TS, AS, and SC, contributed to project design, data analysis, manuscript preparation. AZ, MD, DK, NA, SS, and SC, contributed to data acquisition. NA contributed to manuscript preparation. RH and KK contributed to experimental design. All authors contributed to the article and approved the submitted version.
